# Differential Dynamic Properties of Scleroderma Fibroblasts in Response to Perturbation of Environmental Stimuli

**DOI:** 10.1371/journal.pone.0001693

**Published:** 2008-02-27

**Authors:** Momiao Xiong, Frank C. Arnett, Xinjian Guo, Hao Xiong, Xiaodong Zhou

**Affiliations:** 1 Human Genetics Center, School of Public Health, The University of Texas Health Science Center at Houston (UTHSC-H), Houston, Texas, United States of America; 2 Division of Rheumatology and Clinical Immunogenetics, Medical School, The University of Texas Medical School at Houston, The University of Texas Health Science Center at Houston (UTHSC-H), Houston, Texas, United States of America; 3 Department of Computer Science, Texas A&M University, College Station, Texas, United States of America; University of Glasgow, Germany

## Abstract

Diseases are believed to arise from dysregulation of biological systems (pathways) perturbed by environmental triggers. Biological systems as a whole are not just the sum of their components, rather ever-changing, complex and dynamic systems over time in response to internal and external perturbation. In the past, biologists have mainly focused on studying either functions of isolated genes or steady-states of small biological pathways. However, it is systems dynamics that play an essential role in giving rise to cellular function/dysfunction which cause diseases, such as growth, differentiation, division and apoptosis. Biological phenomena of the entire organism are not only determined by steady-state characteristics of the biological systems, but also by intrinsic dynamic properties of biological systems, including stability, transient-response, and controllability, which determine how the systems maintain their functions and performance under a broad range of random internal and external perturbations. As a proof of principle, we examine signal transduction pathways and genetic regulatory pathways as biological systems. We employ widely used state-space equations in systems science to model biological systems, and use expectation-maximization (EM) algorithms and Kalman filter to estimate the parameters in the models. We apply the developed state-space models to human fibroblasts obtained from the autoimmune fibrosing disease, scleroderma, and then perform dynamic analysis of partial TGF-β pathway in both normal and scleroderma fibroblasts stimulated by silica. We find that TGF-β pathway under perturbation of silica shows significant differences in dynamic properties between normal and scleroderma fibroblasts. Our findings may open a new avenue in exploring the functions of cells and mechanism operative in disease development.

## Introduction

Identifying differentially expressed genes across distinct conditions and clustering co-expressed genes into different functional groups have been general approaches for unraveling molecular mechanisms involved in disease pathogenesis [1]. Although these approaches are valuable for looking at isolated events and their correlations, they do not explain the behavior of a bio-system.

Another approach to deciphering pathogenesis of complex diseases is system thinking. Human complex diseases are believed to arise from malfunction of a specific biological system, rather than from isolated events. It is increasingly recognized that biological systems as a whole are not just the sum of their components but, rather, ever-changing, complex, interacted and dynamic systems over time in response to internal events and environmental stimuli [2]. Cellular functions, such as growth, differentiation, division and apoptosis, and biological phenomena of the entire organisms are not only determined by steady-state characteristics of the biological systems, but also determined by inherent dynamic properties of biological systems. Dynamic properties include stability, transient-response, observability and controllability, which determine how the systems maintain their functions and performance under a broad range of random internal and external perturbations. Similar to differential expression of genes between normal and abnormal tissues, we can also observe the differential dynamic properties of the biological systems across different types of tissues and conditions. Dynamic properties are correlated with the health status of individuals and are of central importance for comprehensively understanding human biological systems and ultimately complex diseases.

The dynamic behavior of most complex biological systems emerges from the orchestrated activity of many components (e.g. genes and proteins) that interact with each other to form complicated biological networks involving gene regulation and signal transduction [3]. The nodes and links together are referred to as networks. This report is a study of gene regulatory network that focuses on dynamic properties of a biological system.

Investigation of dynamic properties of gene networks has three major tasks: development of mathematic models, estimation of the parameters in the models, and dynamic analysis. Mathematical modeling is to use mathematical language to describe the dynamic characteristics of a system [4]. In the past decade, various methods have been developed to model gene networks, including Boolean networks [5]–[7], differential equations and Bayesian networks [8]–[11], and vector autoregressive model [12]. A very powerful approach in modeling complex systems is the state-space approach [13], [14], which is a special subclass of dynamic Bayesian networks. It provides a general framework for the application of dynamic systems theory in the analysis of gene regulation. The state-space approach is the core of modern systems theory. Application of the state-space equations to modeling gene networks allows us to use a large body of methodologies and tools in dynamic systems theory for studying dynamics of gene networks. We use Kalman Filter and Expectation-Maximization (EM) to estimate the parameters in the model [15], [16]. After state-space model of the gene networks is established, the next task is to perform dynamic analysis for the model in response to perturbation of internal and external stimuli. Dynamic analysis attempts to extract inherent features of the systems that capture and describe the behaviors of the system over time under different operating conditions. The most important operating principle of a dynamic system is its stability (i. e., the ability to return to the original state or equilibrium state after perturbation). The concept of stability can be easily illustrated by the example of a marble sitting at the bowl. When the marble is in the bottom of the bowl it is stable. No matter where the marble is pushed, up the side of the bowl or from the bottom of the bowl, after it is released, the marble will finally settle to the bottom of the bowl at the original, stable equilibrium point. However, when the marble is on the top of an inverted bowl, it is unstable. The marble can remain on the top of the bowl only when the forces acting on the marble on the top of the bowl is completely balanced. Any slight perturbation in the marble's steady state will destroy the balance of the marble and cause it to move away from the top of the bowl. This indicates that when the system is in unstable state small perturbation can cause the system move away from the steady-state [17]. The biological systems are in constant change under the influences of genetic and environmental differences. The ability of the systems to maintain the stable states after perturbation and to resist diverse disturbance of the internal and external forces is critical to the viability of living organisms and plays a central role in biology [18], [19]. Consequently, studying stability of biological systems is of great importance for discovering mechanism of complex diseases. Although there has been long history to investigate the stability of biologic systems, to our knowledge, very few studies have been reported on stability of gene networks. Particularly, the relationship between stability of gene networks and status of diseases has not been explored. One of purpose of this paper is to use gene expression data to show that similar to the example of the marble in the bowl, the gene networks will also have stable and unstable states and that unstable gene networks may be associated with the diseases.

Another important property of the dynamic systems is the transient response to disturbance of internal noises and external environmental forces, which measures how fast the systems respond to the perturbation and characterizes damping and oscillation properties of the process in response to the perturbation [13]. Feedback close loops are the basis for maintaining normal function of cells and organisms in the face of internal and external perturbation [19], [20]. The essential feature of the transient response of a feedback closed-loop system largely depends on the location of the closed-loop poles. A simple and popular method for searching the poles of the closed-loop system is the root-locus analysis that plots a curve of the location of the poles of a transfer function of the feedback system over the range of the variable (usually loop gain) to determine whether the system will become unstable or oscillate [13]. The third important property of a dynamic system is controllability. Controllability is defined as the capacity of the system to move from undesired states to certain desired final states in finite time through accessible inputs [21]. Germline or somatic mutations lead to the subsequent transcriptional and translational alterations which will affect the phenotypes of the cells and cause diseases. Therapeutic interventions such as radiation, drug and gene therapy intend to alter gene expressions from an undesired state or abnormal state to a desired or normal state. Theoretic and practical analyses in modern control theory demonstrate that there exist systems which we are not able to change from undesired states to desired states. Now the question arises: are all genetic networks controllable? Can always therapeutic interventions change levels of gene expressions to desired states? Controllability provides answers to these questions. It provides a convenient and sufficient criterion for assessing whether we can change undesired gene expression levels to desired gene expression levels. Controllability describes the ability of biological systems to adapt to the changes of environments and deeply characterizes the internal structure of the system. The controllability of the biological networks may reflect the severity of the disease. Thus, the controllability is a fundamental design principle of biological system.

In summary, stability, transient response, feedback and controllability are basic dynamic properties of the biological systems and are essential to the function of the cells and organisms. As a proof of principle, in this report we investigate the differential dynamic properties of TGF-β pathway in response to perturbation of silica between normal and scleroderma fibroblasts. Scleroderma or systemic sclerosis (SSc) is a typical complex disease in which fibrosis occurs in multiple organs. Although etiopathogenesis is unknown, both genetic and environmental factors are believed to play critical roles. The major source of fibrosis in SSc is over production of collagens from fibroblasts. Fibroblasts obtained from SSc patients appeared to be genetically engineered to produce more collagens and cytokines [22]. Silica exposure is an important environmental risk factor in some cases, which has been found in association with the development and perturbation of SSc [23]. Subcutaneous injections of silica have been reported to induce sclerodermatous skin changes and activation of skin fibroblasts [23]. Therefore, interactions between fibroblasts and silica may represent a magnification of biological events occurring in SSc and/or SSc-like disorders. The biological system of fibroblasts reacting to silica exposure must involve complex regulations and coordination of molecules to maintain their desirable status. Although multiple experiments of the *in vivo* and the *in vitro* response to silica particles have revealed that fibroblasts are activated to produce more collagens and other extracellular matrix (ECM) components [24]–[26], there is a lack of a mathematical model to quantify interactions among the molecules, and to predict dynamic behaviors of this bio-system. The purpose of this report is to use gene expression responses of scleroderma and normal fibroblasts exposed to the stimulus of silica as an example to address the issue of differential dynamic properties of the biological systems in response to perturbation by environments across different conditions. To accomplish this, we first formulate a regulatory network involving TGFBRII, CTGF, SPARC, COL1A2, COL3A1 and TIMP3 as a biological system that is associated with TGF-β signaling, and then apply mathematical methods and computational algorithms from engineering and control theory [13] to perform dynamic analysis of this network for both normal and scleroderma fibroblasts in response to perturbation of environmental Stimuli. Based on the results of dynamic network analysis, we examine the differential dynamic properties of this network between normal and scleroderma fibroblasts and reveal the relationship between the dynamic properties of gene networks and the phenotypes of the cells.

## Results

### State Space Model of a gene network responding to silica

Gene regulation involves a large number of biochemical events. Although kinetic models can be developed for gene regulation [12], [27], [28], they involve many kinetic parameters that are difficult to be estimated from gene expression data with small number of samples. An alternative model of gene expression is a state-space model. It can effectively deal with time invariant or time varying, linear or nonlinear complex systems with multiple inputs and outputs. A state-space model includes three types of variables: input variables, output variables and state variables. A key idea behind state-space model is the concept of the state. The state of a dynamic regulatory system is the smallest set of variables which are referred to as state variables such that the current knowledge of these variables together with the current and future knowledge of the input variables (environments or controls) will completely determine the behavior of the regulatory system. All state variables are hypothetical variables. State variables represent biological forces to regulate transcription of genes, which describe the behavior of gene transcription. Since the mechanisms of gene regulation in the network have still not been well understood, the state variables that determine the regulation may be unknown and hidden in the regulatory process, the concept of state variables is very suitable for description of the regulatory process. The expression levels of genes are output variables and can be observed. The expression levels of the genes are determined by the state variables, which describe states of regulation of the gene expressions.

Previously, we found that the SPARC (secreted protein, acidic, and rich in cysteine) gene is involved in the regulation of extracellular matrix genes such as *COL1A2*, *COL3A1*, *CTGF* and *TIMP3*, and this regulation is associated with activation of the TGF-β pathway [29], [30]. We used this partial TGF-β pathway as an example to illustrate how to perform dynamic analysis of biological networks. This regulatory network was modeled by linear state-space equations defined as:
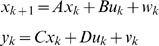
(1)where *x_k_* is the vector of state variables that describes the behavior of gene regulation, but are hidden; *y_k_* is the output vector whose elements denote the measured gene expression levels; *u_k_* is the input vector; *w* and *v* are noises assumed to be white Gaussian noise with zero means and variance *Q* and *R* respectively, and they are independent of each other. The inputs can be any external stimuli that influence gene regulation, things like environmental forces, drugs, proteins, RNAs, or the effects from the genes outside the model. Matrix *A* is called state transition matrix whose elements denote the regulatory strength of one gene on another, *B* is input to state matrix whose elements quantify the regulatory effects of the input variables on the genes of the network, *C* is state to output matrix whose elements quantify the dependence of the measured gene expression levels on the hidden regulatory states, and *D* is input to output matrix whose elements measures the strength of dependence of the observed gene expression levels on the inputs. Matrices *A*, *B*, *C*, *D* and variance matrices *Q* and *R* together make up the parameters of the dynamical system for gene regulatory networks.

We performed experiments on cultured human fibroblasts. We have 5 pairs each of normal and SSc patients' samples. For each sample, we have two replications were perturbed by Silicon. The transcript levels of six genes: *SPARC*, *CTGF*, *COL1A2*, *COL3A1*, *TIMP3* and *TGFBRII were* measured daily from 1 to 5 days. Let *x_1_*, *x_2_*, *x_3_*, *x_4_* and *x_5_* be the expression levels of the *SPARC*, *TIMP3*, *COL3A1*, *CTGF* and *COL1A2*, respectively. Let *u_1_* and *u_2_* be the expression of the *TGFBRII* [31] and 10 µg silica. The estimated state-space model for the normal cell line and SSc are respectively, given by
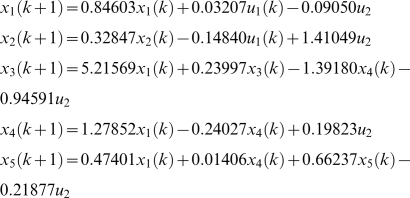
and
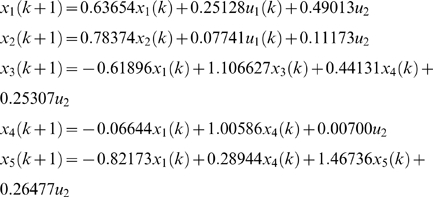



**Figure 1:**
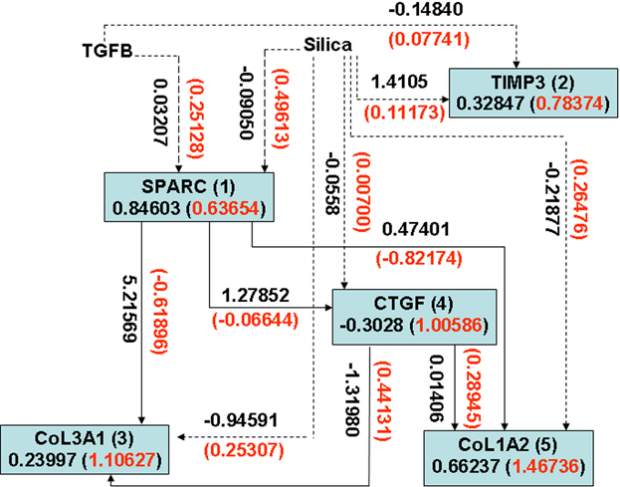
State-space model for the regulatory gene network responding to silica stimulation in cultured human fibroblasts. The numbers next to the edges are the coefficients in the state-space equations for the normal (black color) and SSc (red color) fibroblasts, respectively. The numbers in the boxes denote the mean expression values of the genes in normal (black color) and SSc (red color) fibroblasts.

A graph will be used to represent a genetic network. The nodes in the graph will represent the variables that correspond to the expressions of the genes. The edge between two nodes denotes that two variables are dependent. The number next to edges is the elements of the parameter matrices A, B, C and D in the state-space model. The estimated state-space model is shown in Figure 1 where the numbers next to the edges are the coefficients in the above equation for the normal (black color) and SSc (red color) fibroblasts, respectively. We observe differential regulation of *SPRAC* on *CTGF*, *COL3A1* and *COL1A2*, and *CTGF* on *COL3A1* between the SSc and normal fibroblasts. Figure 1 and above equations also demonstrate that the effects of silica (environmental factor) on *TIMP3*, *COL3A1* and *COL1A2* between the SSc and normal fibroblasts are different. Their coefficients in the state-space equations for the normal fibroblasts are negative, but become positive for the SSc fibroblasts. This implies that perturbation of scleroderma fibroblasts by silica will increase the expressions of *COL1A2* and *COL3A1*. This statement can be supported by early observation that excessive amounts of various collagens mainly type I and type III collagens were generated in the fibroblasts from affected scleroderma skin [32]–[34].

### Stability

The most important dynamic property of gene regulatory networks is concerned with stability. Stability is an organizing principle of gene regulatory networks [35]–[37]. When gene regulatory networks are perturbed, the expressions of the genes in the network will be changed in response to perturbation of environments. There are two possibilities. One possibility is that although the expressions of the genes will be changed after perturbation of environments, they will finally return to their original values or stay at other equilibrium values forever. In this case, regulatory networks will maintain their steady states under perturbation of environments and hence will function normally. Another possibility is that the expressions of the genes after perturbation of the environments will diverge from their original states and never stay at any steady states, which will finally lead to damage and malfunction of the gene regulatory network. Formally, a dynamic system is called stable if their state variables return to, or towards their original states or equilibrium states following internal and external perturbations [38]. The stability of the system is a property of the system itself. One of the methods for assessing the stability of the linear dynamic systems is to analyze eigenvalues of the state transition matrix *A* of the linear dynamic systems. For a discrete linear system, if the norm of all eigenvalues of the transition matrix *A* is less than 1 then the system is stable.

The eigenvalues of the transition matrix *A* of the state-space model for silica responding gene network for normal and SSc fibroblasts are given in Table 1. It indicates that all eigenvalues of the transition matrix *A* for the normal fibroblasts are less than 1, but for SSc fibroblasts, three eigenvalues whose absolute values are larger than 1. Therefore, the examined network for normal fibroblasts after perturbation of silica stimulation are relatively stable, but for SSc fibroblasts are unstable. Unstable gene regulatory or signal transduction networks will lead to erratic changes and malfunction of the whole biological system, which may be the case in the SSc fibroblasts that are associated with dramatic and irregular changes of *COL1A2* and *COL3A1*.

**Table 1:** Eigenvalues of the transition matrix A of the state-space model for the genes in a regulatory network responding to silica in cultured human normal and SSc fibroblasts.

Normal fibroblasts	SSc fibroblasts
0.23997	1.10627
0.66237	1.46736
−0.24207	1.00586
0.84603	0.63654
0.32847	0.78374

### Transient-Response Analysis of Genetic Networks

The dynamic behavior of a system is encoded in the temporal evolution of its states. Cell functions are essentially temporary processes and largely determined by the dynamic properties of the biological systems in the cells. Transient and steady state responses are two steps of the response of a gene network to perturbation of external environments. The transient response of the gene network to perturbation of environments is defined as rapid changes of the expressions of the genes in the network over time which go from their initial states to final states after perturbation of external input [13]. Steady-state response studies the system behavior at infinite time. The transient response of the dynamic systems is also a property of the system itself. The transient response of the gene network to environmental changes characterizes the dynamical process of the gene regulation networks in response to perturbation of environments. It can be used to study damped vibration behavior of the gene network and reveal how fast the gene networks respond to perturbation of environments and how accurately the networks can finally achieve the designed steady-state values. In the previous section we studied stability of the whole gene network, but we did not investigate the stability of the expression of the individual gene in the network. Since the transient response analysis of the gene network will study the dynamic process of the expression of the individual gene, it can be used to assess whether the expression of individual gene after perturbation of environment is stable or unstable. Although many transient response analyses is concerned with delay time, rise time, peak time, maximum overshoot and setting time, in this report, our transient response analysis mainly focuses on investigation of the stability, divergence or oscillation of individual gene expression.

**Figure 2:**
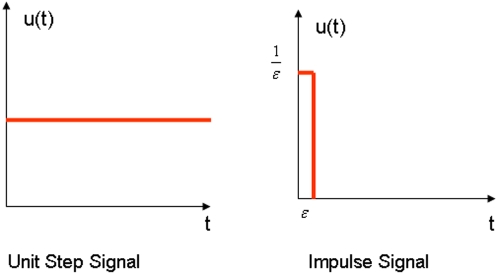
Unit step signal and impulse signal.

Popular methods for investigation of the transient responses of the dynamic systems are to study the time domain characteristics of the system under perturbation of the external signals. The transient response of the dynamic system depends on the input signal. Different input signal will lead to the different transient response of the system. There are numerous types of signal in practice. For the convenience of analysis and comparison, we consider two types of signals: (i) unit-step signal and (ii) unit-impulse signal as shown in the Figure 2.

**Figure 3:**
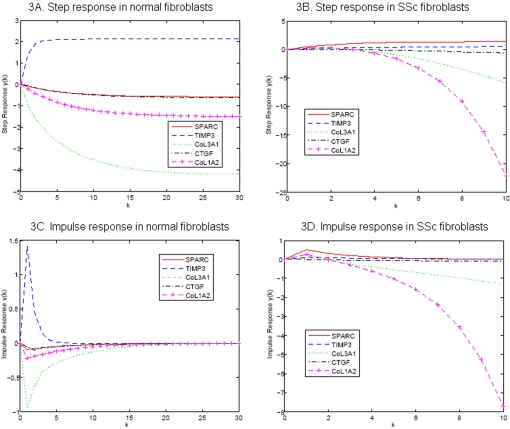
Step response and impulse response of the genes to perturbation of silica in cultured fibroblasts.

For discrete dynamic systems, the transient response of the system is obtained by using the inverse *z* transform method [13]. To investigate the transient response of the genes in the network responding to silica, the silica was taken as input signal. Figures 3A and 3B show transient response of genes to a unit step perturbation of silica for normal and SSc fibroblasts, respectively. Figures 3C and 3D show transient response of genes to an impulse perturbation of silica for normal and SSc fibroblasts, respectively. Figures 3A, 3B, 3C and 3D demonstrate that the transient response of *SPARC*, *TIMP3*, *CTGF* to the perturbation of silica between the normal and SSc fibroblasts are similar, but the transient response of *COL1A2* and *COL3A1* to the perturbation of the silica between the normal and the SSc fibroblasts were dramatically different. The expressions of *COL1A2* and *COL3A1* after perturbation of the silica in normal fibroblasts quickly reach the steady states. However, the expressions of *COL1A2* and *COL3A1* in the SSc fibroblasts after perturbation of silica were unstable and will never reach the steady-state values. This phenomenon suggests that dynamic responses of the expressions of *COL1A2* and *COL3A1* in the SSc fibroblasts to environmental stimuli are irregular.

### Root-Locus Analysis

The performance of the gene networks under the design for stability, time response and reliability can be studied by analysis of their corresponding closed-loop system. The basic features of the stability and transient response of the closed-loop system are largely determined by the location of the closed-loop poles, which in turn is related to the value of the loop gain [13]. The roots of the characteristic equation of a system which is derived from the denominator of transfer function of the closed-loop system are the system's closed-loop poles. In general, the poles are complex variable and can be represented on the complex plane which is called s-plane. (Negative real) poles on the left hand side of the complex plane cause the response to decrease, while poles on the right hand side cause it to increase. Consequently, if the poles of the closed-loop system lie in the left half s plane, the system is stable. If any of these poles lies in the right-hand side of the s-plane, then the system is unstable. In this case, with increasing time, the transient response of the system will increase monotonically or oscillate with increasing magnitude [13]. As the loop gain changes the location of the closed-loop, poles will also changes. A root locus is defined as the locus of the poles of a transfer function of a closed-loop as a specific parameter (generally, loop gain) is varied from zero to infinity. The locus of the poles will be plotted on the complex plane (s-plane) as the system gain is varied on some interval. Since the location of the poles will change as the gain changes a system that is stable for gain K_1_ may become unstable for a different gain K_2_. We often observe that the root-locus will move from the left-hand of the s-plane to the right-hand of the s-plane, which implies that stable system becomes unstable as the system gain changes from one region to another region.

The root-locus plots the locations of the poles of the closed-loop single input and single output system (SISO) as the system gain varies. We use the symbol “x” to denote the poles of the closed-loop SISO and the symbol circle “o” to denote the zeros of the open-loop SISO. If the pole and zero coincide then the symbol ∶ will be used to represent this situation. To study the dynamic behavior of the five genes to respond to the perturbation of silica, we consider the SISO system which takes one of the five genes as the output and silica as the input.

**Figure 4:**
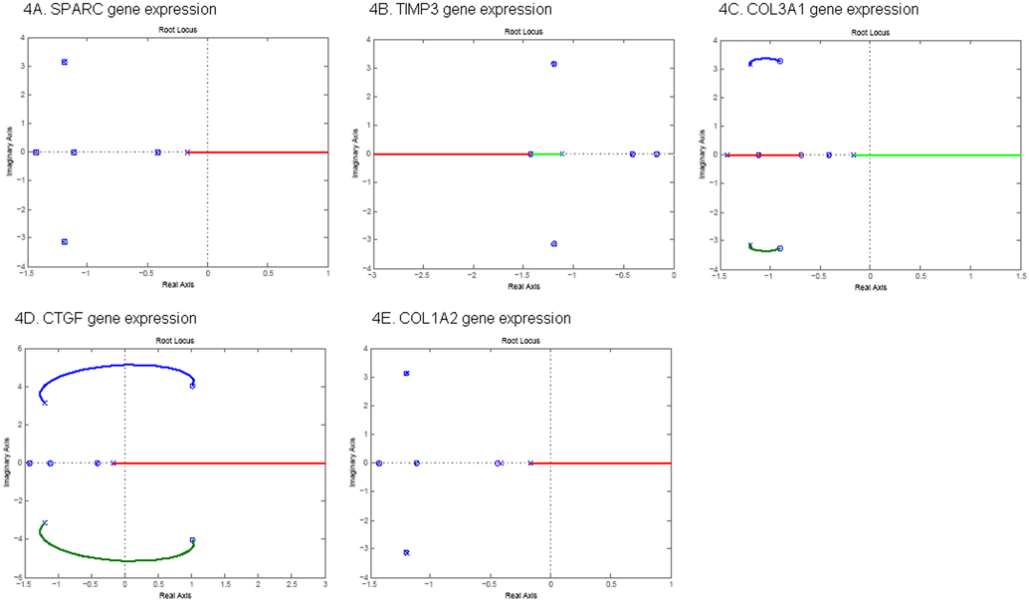
Root-locus of gene expression in normal fibroblasts.

**Figure 5:**
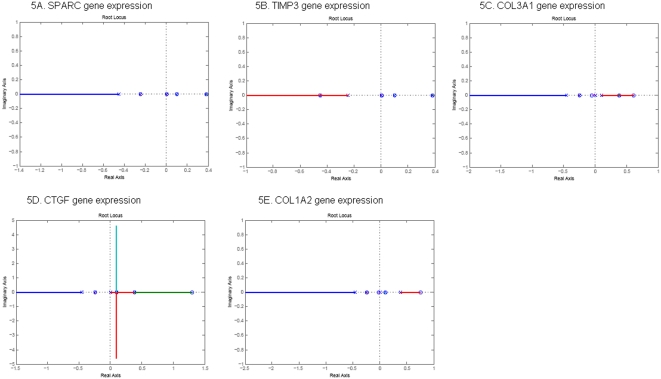
Root-locus of gene expression in SSc fibroblasts.

Figures 4A, 4B, 4C, 4D and 4E, and Figures 5A, 5B, 5C, 5D and 5E show the root-locus plot of *SPARC*, *TIMP3*, *COL3A1*, *CTGF*, and *COL1A2* with silica as the input in the normal and SSc fibroblasts, respectively. We noted that three remarkable features emerged from two panels of the Figures. First, all poles of the closed-loop SISO systems for five genes in the normal cell lines lie in the left hand side of the s-plane, but their corresponding poles in the SSc fibroblasts lie in the right hand side of the s-plane. This indicates that the expressions of all five genes to respond to the disturbance of the environmental silica in normal fibroblasts are stable, but become unstable in the SSc fibroblasts, which confirms the previous stability assessment. Second, although all poles of the closed-loop SISO for five genes in the normal fibroblasts are on the left hand side of the s-plane, the *SPARC*, *COL3A1, CTGF and COL1A2*, each has at least one branch of the root-locus plot which will enter the right-hand side of the s-plane. This indicates that the system becomes unstable as the increasing system gain reaches the some range. This may imply that the regulations of these four genes are sensitive to the changes of the system. Third, the poles and zeros of the SISO on the right hand sides of the s-plane for the *SPARC* and *TIMP3* in the SSc fibroblasts have the same location, i.e., the poles and zeros are cancelled out. This shows that the expressions of *CTGF*, *COL1A2* and *COL3A1* in response to the perturbation of the silica are more unstable than that of *SPARC* and *TIMP3*. Differential regulations of *CTGF*, *COL1A2* and *COL3A1* in response to the perturbation of silica between the normal and SSc fibroblasts may imply that the interactions of these three genes with the silica may be involved in the pathogenesis of the SSc. Forth, these Figures also demonstrate that when normal fibroblasts were changed to SSc fibroblasts, the root-locus will be moved toward the right-half s-plane. Classical control theory indicates that moving of the root-locus toward the right-half s-plane will reduce stability and increase response time of the system.

### Controllability

Changes in expression levels of genes and proteins in the regulatory networks will lead to status transition of the cells from normal cells to abnormal cells. One way to correct molecular changes is to transform cells from an undesirable state to a desirable one by altering gene or protein expressions. Now the question is whether we can use potentially therapeutic interventions to change gene or protein expressions from undesired states to desired states. This important issue can be addressed by examining the controllability of gene regulatory networks.

The fundamental controllability in gene regulation is associated with two questions. The first question is whether an input (therapy) can be found such that the system states can be driven from the undesired initial value to the desired values in a given time interval. The second question is how difficult it may be to change the system from undesired states to the desired states if the system is controllable.

The system (regulatory network) is called controllable, if for any state of the system, there exists a finite time and an admissible control function such that the system can achieve the desired state transition. In other words, the state controllability indicates that we can find an input to change the states from any initial value to any final value within some finite time. The controllability provides a binary information about whether the system is controllable or not, but it does not provide a quantitative measure to quantify the amount of control effort needed to accomplish the control task. It has been recognized that to get into insides of controllability of the system it is indispensible to define a quantity to measure how the system is controllable. In other words, we need to develop a measure to evaluate the amount of control efforts required to change the system from the initial state to the desired state [39]. The test for controllability is that the controllability matrix (see methods) has full rank, i.e., the rank of the controllability matrix is equal to the number of the state variables of the system. To assess how difficult to achieve control goal, we calculated the conditional number of the controllability matrix which measures the degree of difficulty to change the state of the system (or gene expression in our problem) by the external forces such as treatments. The larger the conditional number of controllability matrix, the more control efforts required to accomplish control task.

The rank of controllability matrix of the system in the state-space model of this partial TGF-β pathway under perturbation of silica in both normal and SSc fibroblasts is equal to 5 which is the number of the state variables in the model. Thus, TGF-β pathway is controllable in both normal and SSc fibroblasts. However, the conditional numbers of the controllability matrix of the system for normal and SSc fibroblasts were 80 and 398, respectively, which showed that the conditional number of the controllability matrix for the SSc fibroblasts is much larger than that for normal fibroblasts. Therefore, much more control efforts are required to change gene expressions to desired levels for the SSc fibroblasts than that for the normal fibroblasts. This implies that the controllability of this network between the normal and SSc fibroblasts are differentiable.

## Discussion

In the past, large efforts have been devoted to studying the function of individual genes and static properties of biological pathways. However, the molecular concentrations and activities in living organisms are in constant change as a result of their interactions. The pathogenesis of disease involves evolution and temporal process. The functions of the cells, tissues and entire organisms are not only due to the steady states of the biological pathways, but also due to the dynamic interactions of biological pathways with the external environments. Although investigation of the function of individual genes, proteins and steady-states of the biological pathways is still valuable, it is time to devote more efforts and resources to study dynamic behaviors and properties of the biological pathways. It is dynamic properties that play a central role in giving rise to the function of cells and organisms [40].

To exemplify this principle, we studied the differential dynamic properties of a partial TGF-β signaling network under perturbation of silica between normal and SSc fibroblasts. We took this network as a dynamic system and performed dynamic system analysis. Investigation of differential dynamics of this network between the normal and SSc fibroblasts consisted of three steps. The first step was to use the EM algorithm and Kalman filter to estimate the parameters in the state-space model of this TGF-β signaling network. The second step was to study stability, the transient response and controllability of the system, and to perform root-locus analysis based on the identified state-space model of the gene network. The third step was to assess whether the dynamic properties of this network between the normal and SSc fibroblasts were different.

Our study in dynamic analysis of these gene regulations addressed several remarkable issues. First, the stability analysis may be used as a powerful tool for identifying biological pathways that are associated with the diseases. The stability is one of systems-level principles underlying complex biological pathways [41]. The stability of the system is the ability of the system to return to the equilibrium states after perturbation of the internal and external stimuli. The requirements for stable biological pathways are necessary conditions for the normal operations of the cells and organisms. The unstable biological pathway will inevitably lead to the malfunction of cells or even death of the living organism. Our results showed that a gene network in responding to perturbation of silica is relatively stable in the normal fibroblasts, but unstable in the SSc fibroblasts. This assessment of differential stability of biological pathway between normal and abnormal cells represents a novel approach in study associations of biological pathways with human diseases.

Second, root-locus analysis can provide valuable information for finding genes that show strong differential dynamics between normal and abnormal cells. Not all genes in the unstable pathway show unstable dynamics. Expressions of some genes in the unstable pathway may be stable themselves. Our task is to distinguish the genes that show stable expressions from those show unstable expressions in the unstable pathway. The state transition matrix of the state-space model of the studied gene network in the SSc fibroblasts has three poles that were in the right hand sides of the complex s-plane (Figures 3 and 4), which implies that this network in the SSc fibroblasts is unstable. The zeros of the genes of SPARC and TIMP3 in SISO system coincided with three poles. Therefore, although this gene network was unstable in the SSc fibroblasts, the expressions of the genes of SPARC and TIMP3 were stable. At least one branch of the root locus plots of other three genes (*CTGF*, *COL1A2* and *COL3A1*) were on the right hand sides of the s-plane. This indicates that the responses of the genes of CTGF, COL1A2 and COL3A1 to the perturbation of silica in the SSc fibroblasts were unstable no matter how the system gains were changed. These findings can be confirmed by the transient response analysis of the genes. The poles and zeros of characteristic equations of the SISO systems of the genes in response to the perturbation of internal and external signals are intrinsic properties of the gene regulations and are largely not affected by the expressions of other genes. Unlike the concept of differential expressions of the genes where the differentially expressed genes may be just consequences of differential expressions of other genes lying up in the pathway, the differential stability of the response of the genes to the perturbation of the signal between normal and abnormal tissues may be involved in the pathogenesis of the diseases. Therefore, the genes showing differential stability are supposed to be associated with the diseases. The root-locus analysis and the transient response will provide new tools for identifying the genes that are associated with the diseases. The differential stability and the transient response of the gene in the response to perturbation of the environment between the normal and abnormal cells characterize the interaction between the genes and environments. Therefore, the root-locus analysis and the transient response analysis also provide a powerful tool for detection of the gene-environment interaction.

Third, the controllability of biological pathway is an important property of the system. Germline or somatic mutations lead to the subsequent transcriptional and translational alterations which will finally cause diseases. Therapeutic interventions such as radiation, drug and gene therapy are intended to alter gene expressions from an undesired state to a desired or normal state. Gene regulation is a complex biological system. Theoretic and practical analyses in modern control theory demonstrate that there exist systems which we are not able (or find difficult) to change from undesired states to desired states of gene regulation. Now the question arises: are all biological pathways controllable? Are degrees of controllability of the biological pathways different between normal and abnormal Cells? The controllability measures the ability to move a system around in its entire state space using certain admissible intervention. In this report, we developed a conditional number of controllability matrix, to measure the degree of controllability of the system. Our results show that although a gene expression network responding to silica in both normal and SSc fibroblasts is controllable, the degree of controllability of this regulatory network between the normal and SSc fibroblasts is different. This regulatory network in the SSc fibroblasts has a low degree of controllability. In other words, adjustment of regulation of genes in the network by external intervention in the SSc fibroblasts is more difficult than that in the normal fibroblasts. We suspect that the degree of controllability is correlated with the severity of the diseases. When the diseases are at the initial stages, the biological systems are easy to move from abnormal states to the normal states. The degree of controllability of the system will provide valuable information on the curability of the diseases. Although in the past a number of authors have studied dynamic properties of biological networks, their studies have mainly used kinetic data or artificial data and nonlinear models [28], [35]–[37]. Due to limitation of experiments, many kinetic parameters in the genetic regulation have not been available in practice. Large-scale kinetic analysis of biological networks is infeasible. Here we use gene expressions and linear models to study dynamic properties of genetic networks. The results of this report showed that the dynamic properties of genetic network between normal and abnormal cells were differential.

In summary, dynamic properties of the biological systems are intrinsic system properties. The gene expressions are the phenotype of the cells. Their changes are governed by the hidden dynamic properties of the gene regulatory systems. It is dynamic properties that determine the phenotypes of the cells. This report represents a paradigm shift from the studies of individual components and static properties of the system to the studies of dynamic properties of the system consisting of individual components.

Although the preliminary results are appealing, they suffer from several limitations. First, sample sizes were small. Small sample size will limit the accuracy of the state-space models for biological pathways, which in turn affect estimation of dynamic properties of the systems. No much attention in control theory has been paid to investigation of impact of uncertainty inherent in dynamic systems on dynamic properties of the system. We will treat biological networks as stochastic dynamic system and study dynamic properties of stochastic dynamic systems in the future. Second, the quantities to characterize the dynamic properties are essentially random variables. Their distributions are unknown. We have not developed statistical methods to test significant differences in the dynamic properties of the pathways between the normal and abnormal cells. Third, the relations between the dynamic properties of the genes and their genotypes have not been investigated. Fourth, we have not performed large-scale dynamic analysis of the biological pathways. More theoretical development and large-scale real data analysis for the dynamic properties of the biological pathways are urgently needed.

## Methods

### Dermal fibroblast cultures

Skin biopsies of clinically uninvolved skin (3 mm punch) were obtained from 5 patients with SSc and 5 normal controls after informed consent was granted. All five patients fulfilled American College of Rheumatology criteria for SSc [42]. All five had diffuse skin involvement as defined by LeRoy et al [43], and disease duration of less than five years. Skin biopsies from five normal controls with no history of autoimmune diseases undergoing dermatologic surgery were matched for age (+/− 5 years) and sex. The study was approved by the Committee for the Protection of Human Subjects at University of Texas Health Science Center at Houston.

The skin sample was transported in Dulbecco's Modified Essential Media (DMEM) with 10% fetal calf serum (FCS) (supplemented with an antibiotic and antimycotic) for processing the same day. The tissue sample was washed in 70% ethanol, PBS, and DMEM with 10% FCS. Cultured fibroblast cell strains were established by mincing tissues and placing them into 60 mm culture dishes secured by glass cover slips. The primary cultures were maintained in DMEM with 10% FCS and supplemented with antibiotic and antimycotic.

### Silica stimulation on fibroblasts

The 5th passage of fibroblast strains were plated at a density of 2.5×10^5^ cells in a 35 mm dish and grown until 80% confluence. Culture media then were replaced with FCS–free DMEM containing different doses (1, 5, 10, 25 and 50 µM) of silica particles obtained from Sigma-Aldrich, St Louis, MO. After 24-hour culture at this condition, the fibroblasts were harvested for extraction of RNA. The RNAs were examined with RT-PCR for gene expression of *COL1A2, COL3A1, TGFBRII, CTGF, SPARC* and *TIMP3*. The results from this dose-response assay provided an optimal dose (10 µM) in a time-dependent exposure for fibroblasts, in which 24-, 48-, 72-, 96- and 120-hour exposure of silica were assayed in cultured fibroblasts.

### Quantitative RT-PCR

Quantitative real time RT-PCR was performed using an ABI 7900 sequence detector (Applied Biosystems, Foster City, CA). The specific primers and probes for each gene were purchased through Assays-on-Demand from Applied Biosystems. As described previously (19), total RNA from each sample was extracted from cultured fibroblasts described above using TRIzol reagent (Invitrogen Life Technology) and treated with Dnase I (Ambion, Austin, TX). cDNA was synthesized using Superscript II reverse transcriptase (Invitrogen Life Technology). Synthesized cDNAs were mixed with primers/probes in the 2× Taqman universal PCR buffer, and then assayed on an ABI 7900. The data obtained from assays were analyzed with SDS 2.1 software (Applied Biosystems). The amount of total RNA in each sample was normalized with 18 S rRNA transcript levels.

### State-Space Model and Parameter Estimation

A biological pathway is taken as a dynamic system. The biological system is modeled by linear state-space equations defined as
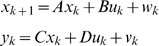
(2)where *x_k_* is a vector of state variables at the time *k* that determine the dynamics of the regulation and unobserved, *u_k_* is a vector of input variables at the time *k* such as drugs, environmental forces, and other state variables that lie outside the model, *y_k_* are observed variables at the time , for example, the gene expressions, *A, B, C,* and *D* are matrices called state matrix, input matrix, output matrix and direct transmission matrix, respectively, *w* and *v* are noises assumed to be white Gaussian noise with zero means and variance *Q* and *R* respectively, and they are independent of each other.

A fundamental and widely applicable parameter estimation method is Maximum Likelihood (ML) method that maximizes the likelihood of the observed data with respect to parameters. However, the state-space models involve unobserved state variables that are unavailable. It makes calculation of the likelihood in the setting of state-space models very difficult. To solve this problem, we use expectation-maximization (EM) Algorithm that is widely used iterative parameter estimation method [15]. Specifically, we first assume that the state variables are available and then calculate the likelihood of both the observed data and hidden state variables which will be maximized with respect to the parameters in the models. After the estimated parameters are in our hands we then specify new state space models using the estimated parameters.

For the convenience of presentation, equation (2) can be rewritten as
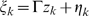
(3)where 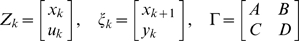 and 
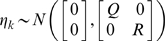
 Then, the conditional density function of *ζ_k_*, given *Z_k_* is given by *N*(Γ*z_k_*, Π), where 
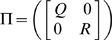
. Assume that the distribution of the initial state is given by *x*_1_∼*N*(*μ*_1_, *P*_1_). Let a sequence of input-output data samples and the state be denoted by
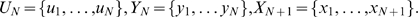
The E-M algorithm for estimation of the parameters in the state-space model of discrete dynamic systems consists of two iterated steps: E-step and M-step. They are summarized as follows [16].

### E-Step

Calculate the expectation of the augmented log-likelihood function of both the observed data and hidden state variables defined as follows:
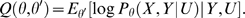
To calculate *Q*(*θ, θ′*), we **first** need to calculate the conditional likelihood function *P_θ_*(*X*, *Y|U*). From the model (3), we have
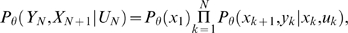
(4)where
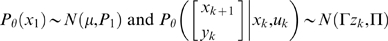
(5)Combining equations (4) and (5), we have
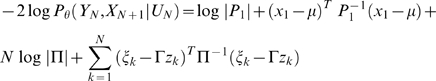
(6)Let
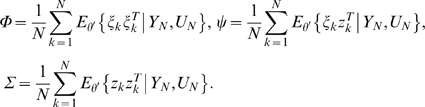
Taking expectation *E_θ_*{.|*Y_N_*, *U_N_*} on both sides of equation (6), we obtain
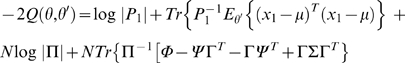
(7)To calculate the matrices Φ and Ψ, we use the following quantities
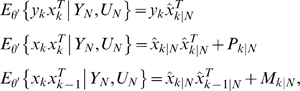
(8)and they can be calculated using Kalman smoother [44]:
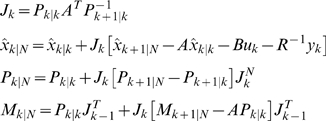
(9)The quantities *x̂_k|k_*, *P_k|k_*, *P_k|k_*_−1_ are calculated by the Kalman filter equations as follows:
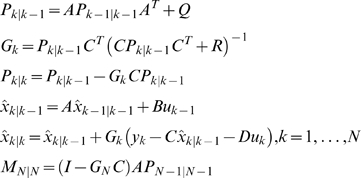
(10)


### M-step

Maximizing the likelihood function defined in equation (7) with respect to parameters yields
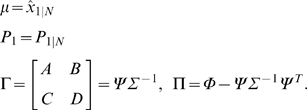
(11)Since the network has structure, which enforces certain parameters in the model to be zeros and leaves others to free to change, we develop constrained EM algorithms.

First we define a matrix product operation of two matrices called Hadamard product, denoted by ○, as element wise product, i.e.
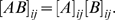
Then, we define a modification of the vector *V,* denoted by [*V*]_mod_, as the vector in which all elements corresponding to the zeros elements in the matrix Γ are deleted. We define a modification of the matrix as the matrix in which if intersection of the row and column corresponds to the zeros elements in the matrix Γ then such row and column are deleted. The equation for estimation of parameters (18) is reduced to
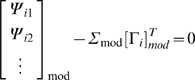


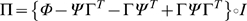



### Transient-Response Analysis

Response of a biological pathway to perturbation of internal and external stimuli has two parts: the transient and the steady state response. The time varying process generated in going from the initial state to the final state in the response to the perturbation of the internal and external stimuli is called transient response. Steady-state response studies the system behavior at infinite time. Transient-response analysis of biological pathways can be used to quantify their dynamics. It can reveal how fast the biological pathways respond to perturbation of environments and how accurately the pathways can finally achieve the desired steady-state values. It can also be used to study damped vibration behavior and stability of the biological pathways.

The transient response of the dynamic systems depends on the input signals. Different signal will cause different response. There are numerous types of signal in practice. For the convenience of comparison, we consider two types of signals: (i) unit-step signal and (ii) unit-impulse signal as shown in Figure 2.

The transient response of dynamic systems can be studied by transfer function that is used to characterize the input-output relationships of a linear, time-invariant, differential equation system. The transfer function is defined as the ratio of the Laplace transform of the output to the Laplace transform of the input under the assumption of zero initial conditions. The transfer function of the response of the biological pathway to unit-step and unit-impulse input signals are given by 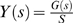 and *Y*(*s*) = *G*(*s*) respectively, where *G*(*s*) is the transfer function of the biological pathway. The transient-response analysis of the biological pathway can be performed by inverse Laplace transformation. We performed the transient-response analysis with MATLAB [13].

### Stability Analysis

The most important dynamic property of biological pathways is concerned with stability. Dynamic systems are called stable if their variables such as gene expressions return to, or towards, their original or equilibrium states following internal and external perturbations. For any practical purpose, the biological pathways must be stable. Unstable gene regulations will lead to the malfunction or even the death of the cells. A biological pathway will remain at steady state until occurrence of external perturbation. Depending on dynamic behavior of the system after perturbation of environments, the steady-states of the system are either stable (the system returns to the initial state or changes to other steady-states) or unstable (the system leaves the initial equilibrium state).

One of the methods for assessing the stability of the linear dynamic systems is to analyze eigenvalues of the state transition matrix *A* of the linear dynamic systems. For a continuous linear dynamic system, if real parts of all eigenvalues of the transition matrix *A* are strictly negative then the system is stable. For a discrete linear system, if the norm of all eigenvalues of the state transition matrix *A* is less than 1 then the system is stable.

### Root-Locus Analysis

Open and close loop poles and zeros largely determine the stability and performance of the open and close systems. They provide valuable information on how to improve stability and transient response of the systems. Root-locus analysis, in which the roots of the characteristic equation of the closed-loop system are plotted for all values of a system parameter, is a powerful tool for study and design of dynamic pathway. The loop gain is often chosen to be the parameter. Varying the gain value will change the location of the closed-loop poles.

**Figure 6:**
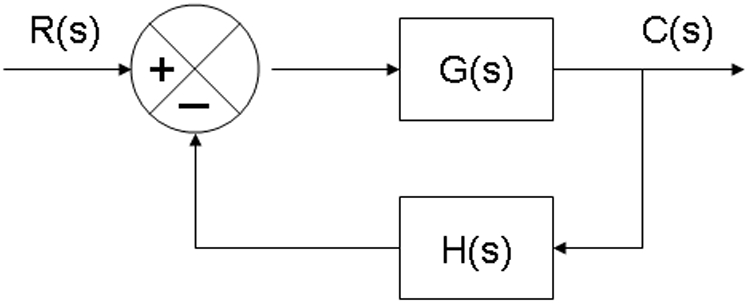
Scheme of a SISO system

Consider a SISO system that consists of a gene regulator and an input to the gene regulator shown in Figure 6. The transfer function of the closed-loop system is given by
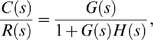
which implies the following characteristic equation of this closed-loop system:
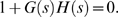
(12)


In general, *G*(*s*) *H*(*s*) involves a gain parameter *K*. A plot of the points in the complex plane satisfying the characteristic equation (12) is the root locus. As the gain parameter changes the root locus will plot a curve in the complex s-plane. A simple method for plotting root-locus has been developed by W. R. Evans [29]. In this report, we use MATLAB to generate root-locus plots [13].

### Controllability

A dynamic system is called controllable, if there is an admissible control function, which can change the system from any given initial state to any finite state or to the origin of the state space in the finite time. Define the controllability matrix of the system as *H* = [*B*, *AB*, …, *A^n^* ^−1^ *B*], where *A* and *B* are the state transition matrix and input to the state matrix in the linear dynamic system, respectively. If rank (*H*) = *n*, i.e., the rank of the controllability matrix is equal to the number of the state variables of the system, then the genetic network is completely controllable.

We use the condition number of the controllability matrix to measure the degree of the controllability of the system. The condition number of the controllability matrix is defined as [45]

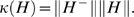
where *H* ^−^ is a generalized inverse of the matrix *H* and ||.|| denotes a matrix norm. This can be justified by the following arguments. The general solution of the discrete linear system is given by [13]

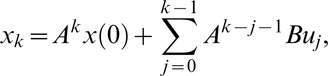
(13)By definition, if the system is controllable, then at some time *t_k_*, we have *x_k_* = 0, which implies that
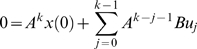
(14)


Equation (14) can be reduced to
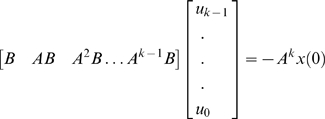
Or
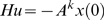
(15)where *u* is a control vector. Solving the equation (15) yields
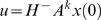
(16)The norm of the control vector represents the amount of control efforts required to change the states from initial value to the desired value and hence measures the degree of the controllability. From equation (16), we note that the norm of the control vector ||*u*|| is proportional to the condition number: *κ*(*H*) = ||*H* ^−^||||*H*||. Therefore, we can use the condition number of the controllability matrix to measure the degree of controllability.
